# Revealing the link between gut microbiota and brain tumor risk: a new perspective from Mendelian randomization

**DOI:** 10.3389/fcimb.2024.1404745

**Published:** 2024-08-06

**Authors:** Jianyu Yang, Jietao Lu, Yuhan Dong, Youdong Wei, Michael Christian, Junmeng Huang, Haiyan Kuang, Du Cao

**Affiliations:** ^1^ Department of Neurology, The First Affiliated Hospital of Chongqing Medical University, Chongqing, China; ^2^ Department of Clinical Nutrition, Bishan Hospital of Chongqing, Bishan Hospital of Chongqing Medical University, Chongqing, China; ^3^ Department of Neurology, The Second People’s Hospital of Banan District, Chongqing, China

**Keywords:** brain tumors, causal relationship, FinnGen, gut microbiota, Mendelian randomization

## Abstract

**Background:**

Recent studies have shown that gut microbiota may be related to the occurrence of brain tumors, but direct evidence is lacking. This study used the Mendelian randomization study (MR) method to explore the potential causal link between gut microbiota and brain tumors.

**Method:**

We analyzed the genome-wide association data between 211 gut microbiota taxa and brain tumors, using the largest existing gut microbiota Genome-Wide Association Studies meta-analysis data (n=13266) and combining it with brain tumor data in the IEU OpenGWAS database. We use inverse-variance weighted analysis, supplemented by methods such as Mendelian randomization-Egger regression, weighted median estimator, simple mode, and weighted mode, to assess causality. In addition, we also conducted the Mendelian randomization-Egger intercept test, Cochran’s Q test, and Mendelian randomization Steiger directionality test to ensure the accuracy of the analysis. Quality control includes sensitivity analysis, horizontal gene pleiotropy test, heterogeneity test, and MR Steiger directionality test.

**Result:**

Our study found that specific gut microbial taxa, such as order Lactobacillales and family Clostridiaceae1, were positively correlated with the occurrence of brain tumors, while genus Defluviitaleaceae UCG011 and genus Flavonifractor were negatively correlated with the occurrence of brain tumors. The Mendelian randomization-Egger intercept test showed that our analysis was not affected by pleiotropy (P>0.05).

**Conclusion:**

This study reveals for the first time the potential causal relationship between gut microbiota and brain tumors, providing a new perspective for the prevention and treatment of early brain tumors. These findings may help develop new clinical intervention strategies and point the way for future research.

## 1 Introduction

The incidence of brain tumors may not be very high, but the survivability rate is very small. This occurs due to the treatment complexity, mainly related to the inability to remove normal brain tissue and the rapid spread of malignant tumors (Zhao et al., 2023). Brain tumors become one of the leading causes of cancer death, especially in children (Lago et al., 2023). Cancer mortality rates from brain tumors are 30% and 20% respectively in children and adults, with meningiomas being the most common (39.0%), followed by pituitary tumors (17.1%) and glioblastoma (14.3%) (McNeill, 2016). In 2015, brain tumors were estimated to account for 1.4% of new cancer diagnoses and 2.6% of cancer deaths (McNeill, 2016).

In recent years, with the advancement of the connection between microbiology and neurology, rising evidence has shown that gut microbiota plays a key role in the normal physiological activities and pathological changes of the brain through the brain-gut axis (Cryan et al., 2019). An in-depth study of the brain-gut axis revealed that it is a complex bidirectional communication network between the gut and the central nervous system that involves multiple pathways. Neurotransmitters and microbial metabolites affect the central nervous system through this network, thereby affecting behavior, memory, learning, and movement. These actions may lead to a variety of neurological diseases (Rutsch et al., 2020). Research showed that gut microbiota play a fundamental role in priming and regulating the immune system. It has been observed that the gut microbiota will change significantly when brain tumors occur (Rutsch et al., 2020; Lin et al., 2023). For example, the number of *Firmicutes* and their ratio to *Bacteroides, Verrucomicrobia*, and *Akkermansia* decreased, while *Enterobacteriaceae* increased in meningioma patients (Lin et al., 2023). These changes may affect the human immune system and intestinal ecology and are potentially related to the occurrence of brain tumors. In addition, since the blood-brain barrier forms a special immune environment of the brain, immune system regulation is crucial to prevent and develop brain tumors (Rong et al., 2022).

With the in-depth study of the brain-gut axis, the causal relationship between gut microbiota and brain tumors has become a trending topic in research. Mendelian randomization (MR) explores the causal relationship between genetic variation and outcome by analyzing genetic variation as an exposure factor. MR utilizes single nucleotide polymorphisms (SNPs) as instrumental variables (IVs) to infer this relationship (Greenland, 2018). MR methods have been widely used to study the relationship between gut microbiota and various neurological diseases, such as epilepsy, stroke, neurodegenerative diseases, etc.) (Ning et al., 2022; Meng et al., 2023; Zeng et al., 2023).

## 2 Methods

### 2.1 Study design and the assumption of MR

**Figure 1:**
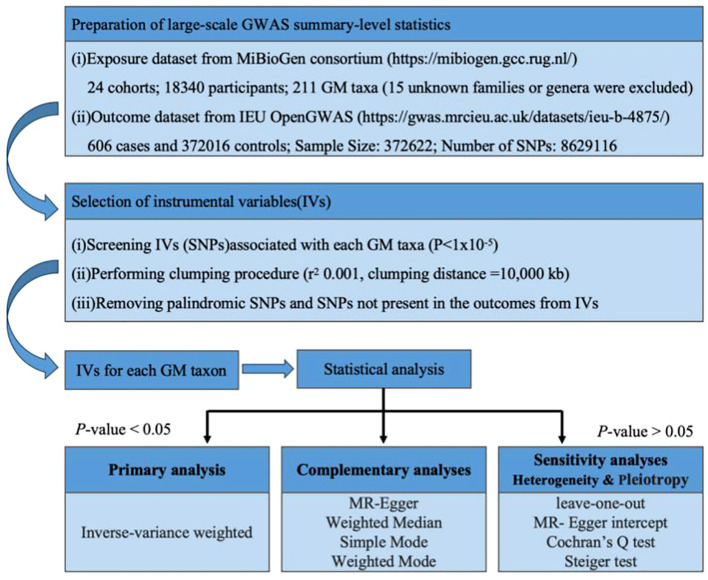
Study Design and Workflow Summary.

The basic principle of MR is to use genetic variants associated with exposure and outcome as instrumental variables (IVs) to infer whether there is a causal association between the two. The basic steps include: obtaining GWAS summary data, SNP screening and evaluation, statistical analysis, and quality inspection. The accuracy of MR analysis is based on the satisfaction of the following three core assumptions (Didelez and Sheehan, 2007): (1) IVs need to be closely related to exposure; (2) IVs have nothing to do with confounding factors that affect “exposure-outcome”; (3) IVs only Data Sources by exposure without affecting them through other means. Because MR-Egger regression is an effective tool for detecting and adjusting horizontal pleiotropy in instrumental variables, through this step we are able to assess whether genetic variants directly affect outcome variables through other pathways besides the exposed variables. **Figure 1** summarizes the overall study design and workflow.

### 2.2 Data source

Data related to gut microbiota come from the MiBioGen study (https://mibiogen.gcc.rug.nl/menu/main/home) (Kurilshikov et al., 2021). The MiBioGen study consists of 24 population-based cohorts with a total of 18,340 participants. The GWAS data set included a total of 211 gut microbiota taxa, of which 15 were unknown families or genera and were excluded, leaving 196 microbial taxa for MR analysis.

Summary statistics for brain tumors are from the IEU OpenGWAS database (https://gwas.mrcieu.ac.uk/datasets/ieu-b-4875/). The sample size of cases (ncase) is 606, the sample size of the control group (ncontrol) is 372016, the total sample size (Sample Size) is 372622, and the number of SNPs (number of SNPs) is 8629116. See **Supplementary Table 1** for details.

### 2.3 screening and evaluation of SNPs (IV selection)

To ensure the inclusion of appropriate IVs, the specific steps for selecting IVs in this study are as follows: (1) In the obtained exposed GWAS database, select exposure-related SNPs based on P<1 × 10^−5^ (McDaid et al., 2017), the reason for choosing this threshold is to include more IVs and improve the accuracy and testing efficiency of MR analysis; (2) To ensure that the selected IVs are independent of each other, this study set the linkage disequilibrium standard as R*^2^* < 0.001, distance = 10,000 kb to exclude SNPs with linkage disequilibrium (Slatkin, 2008); (3) SNPs related to exposure are matched in the GWAS database of outcomes, and the screening condition is P<5 × 10^−5^; (4) Integration of two sets of data: use the “harmonize_data” function to unify the data based on the statistical parameters of the same sites in the GWAS results of exposure and outcome. During this process, the palindromic sequence was deleted (the palindromic sequence is an SNP with the same base sequence in the forward and reverse strands of DNA but in opposite directions), and finally, a new data framework combining exposure and outcome was obtained (Hartwig et al., 2016). (5) Use the F statistics to evaluate the strength of IVs, $F=\frac{\beta^{2}}{se^{2}}$ (Rosa et al., 2019; Chen et al., 2021; Feng et al., 2022). IVs with F<10 are considered weak instruments (Burgess et al., 2017) and are excluded from the subsequent MR analysis.

### 2.4 Statistical analysis

After determining the final included IVs according to the above-mentioned screening process, MR analysis begins. This study uses 5 methods to estimate causal effects: inverse-variance weighted (IVW), MR-Egger regression, weighted median estimator (WME), simple mode (SM), and weighted mode (WM) (Wootton et al., 2018; Hwang et al., 2019). Since the IVW method has higher testing efficiency than the other four MR methods (Lin et al., 2021), this study uses the IVW method as the preferred causal effect estimation method.

Quality control includes sensitivity analysis, horizontal gene pleiotropy test, heterogeneity test, and MR Steiger directionality test (Bowden et al., 2018). (1) Sensitivity analysis uses the “leave-one-out” function in the R package to reanalyze the results by eliminating IVs one by one to compare the impact of each SNP on the results. The results will be in the form of a forest plot. (2) Horizontal gene pleiotropy testing evaluates whether IVs affect outcomes through pathways other than exposure. This test performs MR-Egger regression and returns its intercept, calculated using the “mr_pleiotropy_test” function in the “TwoSampleMR” package. The horizontal gene pleiotropy test result will be found to be insignificant if P>0.05. A non-statistically significant pleiotropy test means there is no need to consider the influence of gene pleiotropy at this time (Cho et al., 2020). (3) The heterogeneity test uses Cochran’s Q test method to evaluate the possible bias in causal effect estimation caused by SNP measurement errors caused by different analysis platforms, experimental conditions, analysis populations, etc. The Q test is suitable for large sample data and is calculated using the “mr_heterogeneity” function in the “TwoSampleMR” package. When the test result shows P>0.05, the impact of heterogeneity on the research results can be ignored at this time (Gill, 2020). (4) To ensure that the direction of the research results is consistent with the research design, this study conducted the MR Steiger directionality test. The MR Steiger test was used to test whether the hypothesis that exposure causes the outcome is valid. Use the “directionality_test” function in the “TwoSampleMR” package. When the result is displayed as TRUE, it means that the direction of the causal relationship is consistent with the hypothesis (Hemani et al., 2018).

All analyses were performed using TwoSampleMR (version 0.5.7), MendelianRandomization (version 0.8.0), and MRPRESSO packages (1.0) in R software 4.3.1 (https://www.R-project.org).

## 3 Result

### 3.1 Two-sample MR study

**Figure 2:**
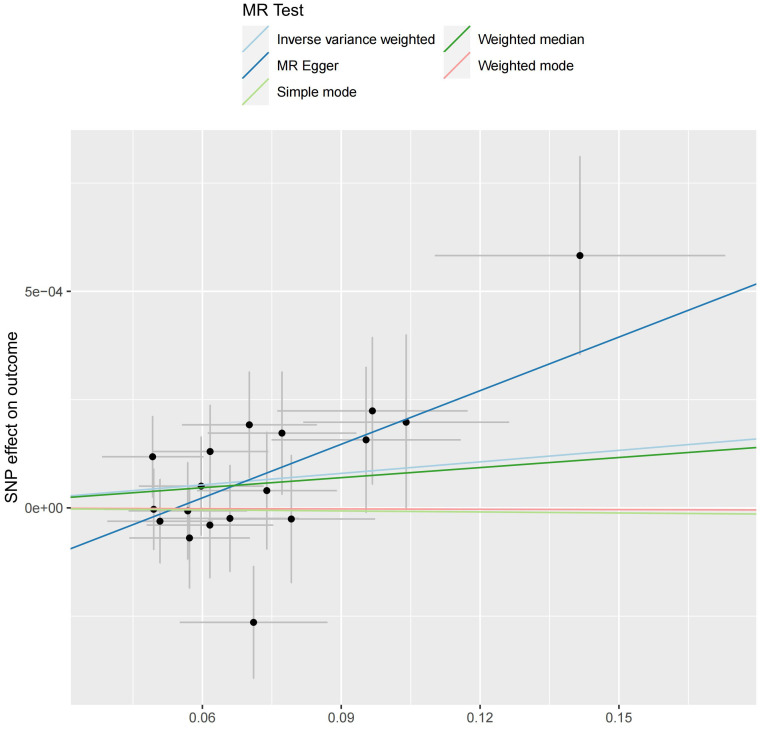
The SNP effect of the *genus Fusicatenibacter* on the outcome in different ways.

**Figure 3:**
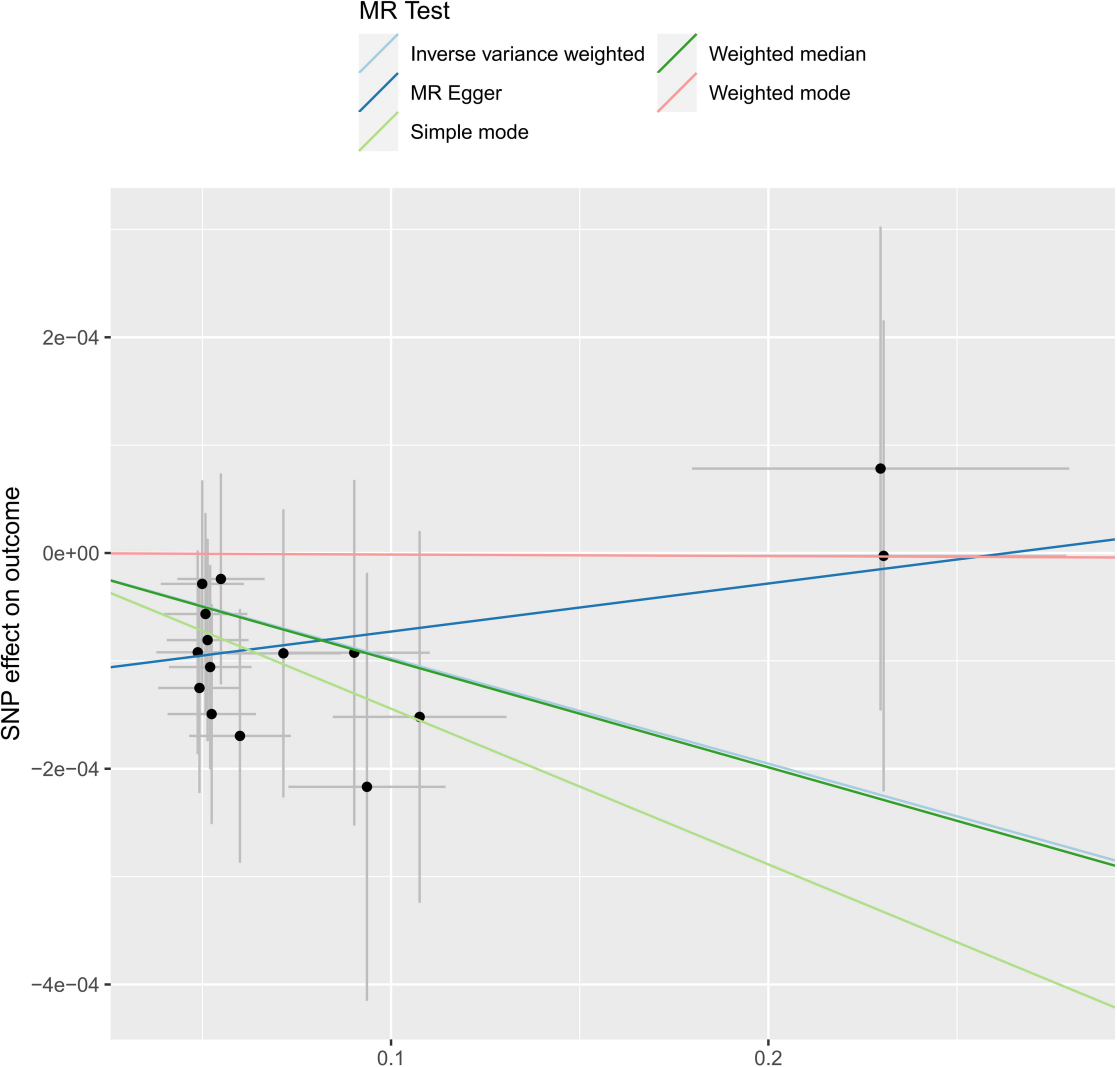
The SNP effect of the *genus Lachnospiraceae* NK4A136 group on the outcome in different ways.

We analyzed the association between gut microbiota and brain tumor risk using an MR methods. IVW results show that *order Lactobacilales* (regression coefficient b=0.001214, P value=0.009952), *family Clostridiaceae*1 (b=0.001050, P value=0.042044), *family Oxalobacteraceae* (b=0.000605, P value=0.022776), *genus Clostridium sensu stricto1* (b= 0.001085, P value=0.039446), and genus Fusicatenibacter (b=0.000885, P value=0.047871) were associated with a slightly increased risk of brain tumors. Meanwhile, *genus Defluviitaleaceae* UCG-011 (b=-0.001044, P value=0.014386), *genus Flavonifractor* (b=-0.001267, P value=0.043292), and *genus Lachnospiraceae* NK4A136 group (b=-0.000976, P value=0.017517) are associated with a decreased risk of brain tumors. However, given the potential confounding factors, the potential invalidity of instrumental variables, and the complexity of the relationship between the gut microbiotas (*genus Fusicatenibacter* and *genus Lachnospiraceae* NK4A136 group) and brain tumor risk, we observed different method produces inconsistent regression coefficient directions. Therefore, when interpreting the causal link between the *genus Fusicatenibacter* and *genus Lachnospiraceae* NK4A136 group and brain tumor risk, further consideration should be implemented and a comprehensive evaluation combined with evidence from other biological and epidemiological studies should be considered. **Figures 2**, **3** show the relationship between microbes and outcomes under different methods (IVW, MR-Egger regression, WME, SM, WM).

### 3.2 Quality control

The leave-one-out analysis method showed that no SNP in *order Lactobacillales, family Clostridiaceae1, family Oxalobacteraceae, genus Clostridium sensu stricto1, genus Defluviitaleaceae UCG-011, and genus Flavonifractor* had a dominant effect on the overall evaluation, as displayed in **Supplementary Figure 1**. In the MR-Egger intercept test, all P values were >0.05, indicating that the results were not statistically significant and gene pleiotropy’s impact on the research results should not be considered. The heterogeneity test results showed that in the IVW method, all P values were >0.05, indicating that the results were not statistically significant, and heterogeneity’s impact on the research result should not be considered. The MR Steiger directionality test result is TRUE, indicating that the results were consistent with the expected direction. The results of the three tests are shown in **Supplementary Table 2**.

## 4 Discussion

In this study, we performed a two-sample MR analysis of brain tumor statistics from the MiBioGen study and the IEU OpenGWAS database, aiming to evaluate the causal link between gut microbiota and brain tumors. We discovered six potential causal relationships between gut microbes and brain tumors. To our knowledge, our study is the first to use MR analysis to explore potential causal relationships between gut microbiota and brain tumors. Research in recent years has increasingly shown that gut microbiota interacts with the central nervous system and may be related to the occurrence of certain neurological diseases. Research shows that the brain-gut axis plays an important role in tumor proliferation, invasion, apoptosis, autophagy, and metastasis (Ning et al., 2022). Neurotransmitters produced by the gut microbiota bind to tumor cell receptors and produce different biological effects. This occurs in many common tumors: gastrointestinal tumors, lung tumors, and liver tumors, and has been confirmed to be closely related to gut microbiota (Budden et al., 2017; Meng et al., 2018; Jia et al., 2019; Gu et al., 2023). Recent studies have also shown that gut microbiota can promote the development of cancer by affecting the balance between host cell proliferation and apoptosis and affecting the immune system (Ning et al., 2022). Defects in the immune system are one of the main causes of brain tumors (McNeill, 2016). Gut microbiota can mediate neurophysiological processes by regulating the development and function of microglia and astrocytes, thereby participating in the formation of brain inflammation and damage (Fung et al., 2017). Studying the potential causal relationship between gut microbiota and brain tumors, as well as its impact on brain behavior and function, will provide new ideas for the future treatment and prevention of brain tumors.

We found six gut microbiota species associated with brain tumor risk: *order Lactobacilales, family Clostridiaceae1, family Oxalobacteraceae, genus Clostridium sensu stricto1, genus Defluviitaleaceae* UCG-011, and *genus Flavonifractor*. Although the causal association is weak, the results had reference value and showed a prospective in-depth analysis in this direction. The study by Hacioglu et al. found that the abundance of *Firmicutes* and the ratio of *Firmicutes/Bacteroides* in patients with acromegaly decreased, and *Bacteroides* increased (Hacioglu et al., 2021), and the most common cause of acromegaly is pituitary adenoma (Giantini-Larsen et al., 2022),which supports the conclusion that *genus Defluviitaleaceae* UCG011 and *genus Flavonifractor* are inversely related to brain tumors. At present, the mechanism of interaction between brain tumor occurrence and gut microbiota is still unclear (Kim et al., 2016). The study found that the gut microbiota changes in mouse glioma models and patients have consistent trends, and are affected by the chemotherapy drug temozolomide (Patrizz et al., 2020). Several recent studies have also explored the relationship between gut microbiota and gliomas (Cui et al., 2024; Ishaq et al., 2024).Therefore, the interactions among multiple microorganisms deserve further study.

To our knowledge, this is the first time that MR analysis has been used to explore the potential relationship between brain tumors and gut microbiota. This method eliminates confounding variables, reverses the causal inference process, and reduces the impact of common confounding factors. MR analysis is based on large, publicly available GWAS databases, significantly reducing experimental costs. Following the analysis results, we employed five quality control methods to ensure the accuracy and reliability of causal relationships. Our study had several limitations. Since the number of SNPs consistent with P<5 × 10^−8^ was limited, we selected SNPs with P<1× 10^−5^ as instrumental variables (IV) to conclude more candidates. The data we used was limited to European ancestry, hence there could be potential bias related to ethnicity and demographics.

## 5 Conclusion

In summary, this study comprehensively explores the potential causal link between gut microbiota and brain tumors. Six types of gut microbiotas were found to be related to the risk of brain tumors. These findings provide new directions and ideas for the prevention and treatment of brain tumors in the future.

## Data availability statement

The original contributions presented in the study are included in the article/**Supplementary Material**. Further inquiries can be directed to the corresponding author.

## Ethics statement

The studies involving humans were approved by Ethics Committee of The First Affiliated Hospital of Chongqing Medical University. The studies were conducted in accordance with the local legislation and institutional requirements. The participants provided their written informed consent to participate in this study.

## Author contributions

DC: Writing – review & editing. JY: Data curation, Formal analysis, Methodology, Writing – original draft. JL: Data curation, Formal analysis, Methodology, Writing – original draft. YD: Methodology, Validation, Writing – original draft. YW: Investigation, Methodology, Writing – review & editing. MC: Data curation, Writing – original draft. JH: Supervision, Validation, Writing – original draft. HK: Data curation, Writing – original draft.

## Supplementary material

Supplementary Table 1 Summary of Data Used in the Analysis.

Supplementary Table 2 Approximate GM to P-value of Significance.

Supplementary Figure Sensitivity Analysis Leave-One-Out Result.
