# Supplementary material for: Revealing the link between gut microbiota and brain tumor risk: a new perspective from Mendelian randomization
Source: Front Cell Infect Microbiol. 2024 Aug 6;14:1404745. doi: 10.3389/fcimb.2024.1404745 (PMC11333460; doi:10.3389/fcimb.2024.1404745)
Supplement: Supplementary Table 1 — Summary of Data Used in the Analysis. [file Table_1.pdf]

Supplementary Table 1. Summary of Data Used in the Analysis

Exposure and Outcome	Sample Size	Sample Source	Download Link
Gut Microbiota	18,340 participants	Mixed Population	https://mibiogen.gcc.rug.nl
Brain Tumors	606 cases<br>372016 controls	European	https://gwas.mrcieu.ac.uk/datasets/<br>ieu-b-4875/
