# Supplementary material for: Revealing the link between gut microbiota and brain tumor risk: a new perspective from Mendelian randomization
Source: Front Cell Infect Microbiol. 2024 Aug 6;14:1404745. doi: 10.3389/fcimb.2024.1404745 (PMC11333460; doi:10.3389/fcimb.2024.1404745)
Supplement: Supplementary Table 2 — Approximate GM to P-value of Significance. [file Table_2.pdf]

Supplementary Table 2. Approximate GM to *P*-value of Significance

Exposure	Heterogeneity Test			Gene Pleiotropy Test		MR SteigerTest	
	Method	Q Value	*P* Value	Egger Intercept	*P* Value	Directionality	*P* Value
order Lactobacillales	IVW	10.458	0.575	-0.00017	0.080	TRUE	6.47E-55
family Clostridiaceae1	IVW	2.582	0.978	4.55E-05	0.688	TRUE	9.92E-42
family Oxalobacteraceae	IVW	5.560	0.960	2.72E-05	0.849	TRUE	3.50E-63
genus Clostridium sensu stricto1	IVW	5.632	0.465	3.44E-05	0.804	TRUE	3.65E-29
genus Defluviitaleaceae UCG-011	IVW	6.284	0.506	8.11E-05	0.628	TRUE	1.33E-36
genus Flavonifractor	IVW	1.260	0.868	6.38E-05	0.780	TRUE	1.59E-64
