# Supplementary figures and images for: Revealing the link between gut microbiota and brain tumor risk: a new perspective from Mendelian randomization
Source: Front Cell Infect Microbiol. 2024 Aug 6;14:1404745. doi: 10.3389/fcimb.2024.1404745 (PMC11333460; doi:10.3389/fcimb.2024.1404745)

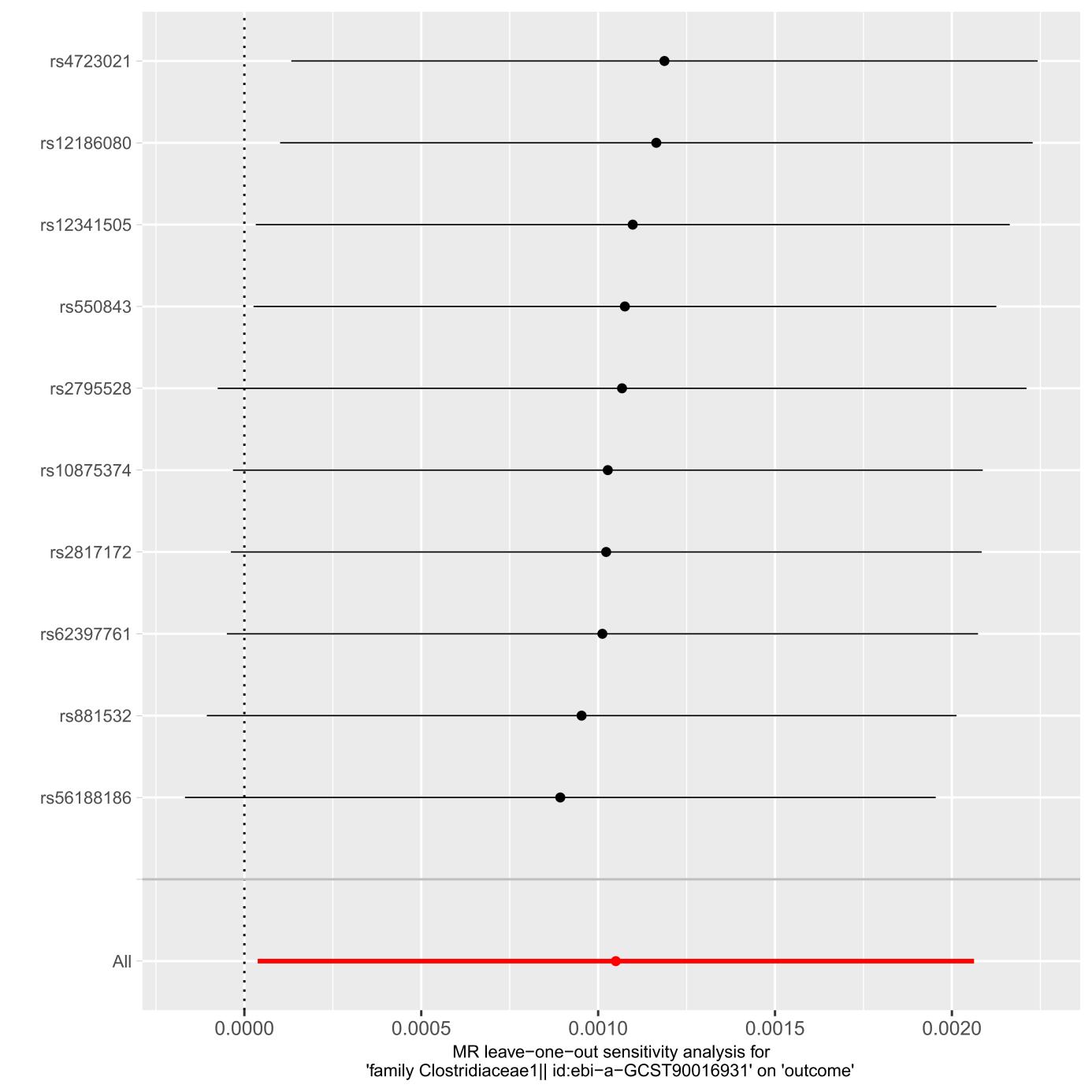

Supplement: Supplementary Figure — Sensitivity Analysis Leave-One-Out Result. [file Image_1.jpg]

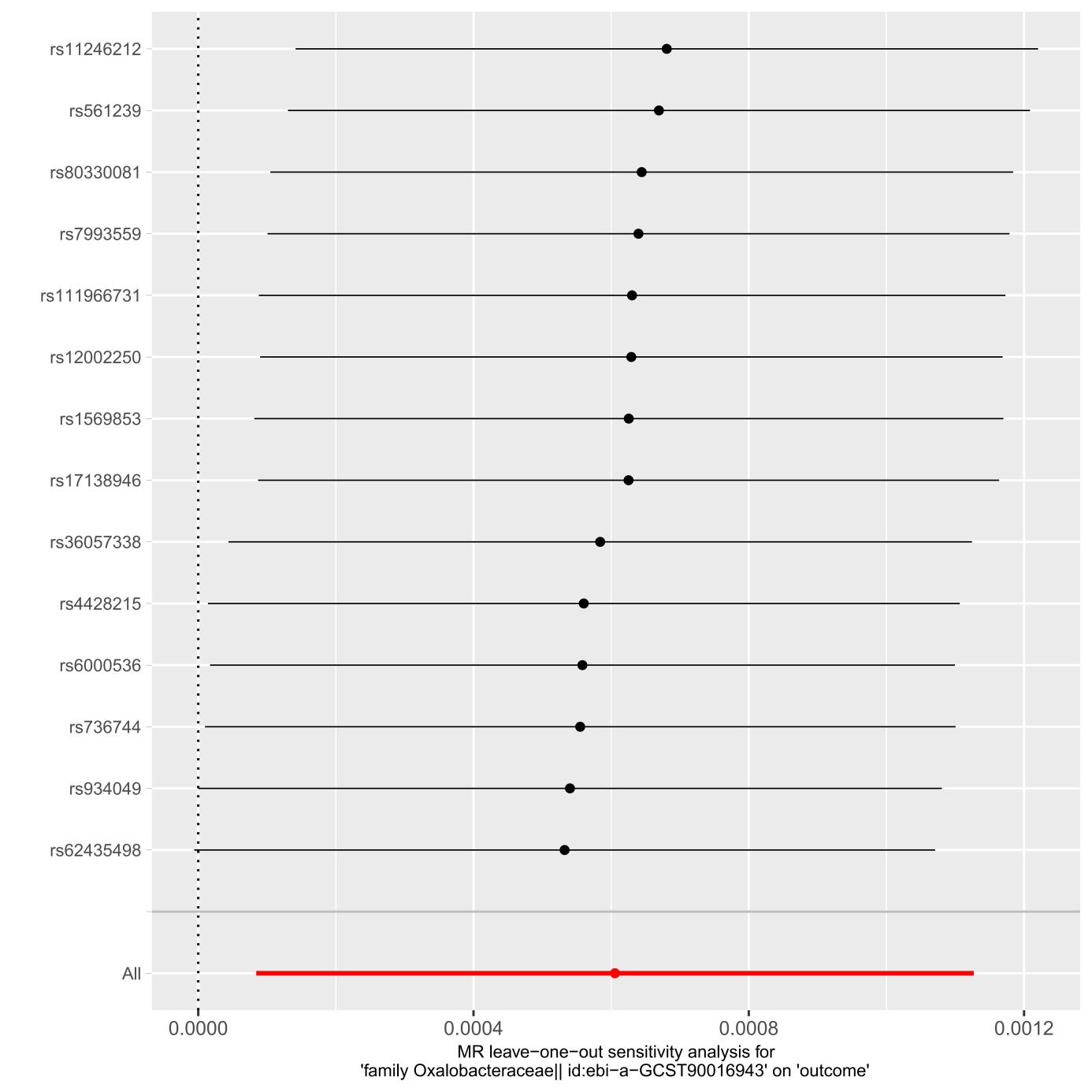

Supplement: Supplementary file 4 [file Image_2.jpg]

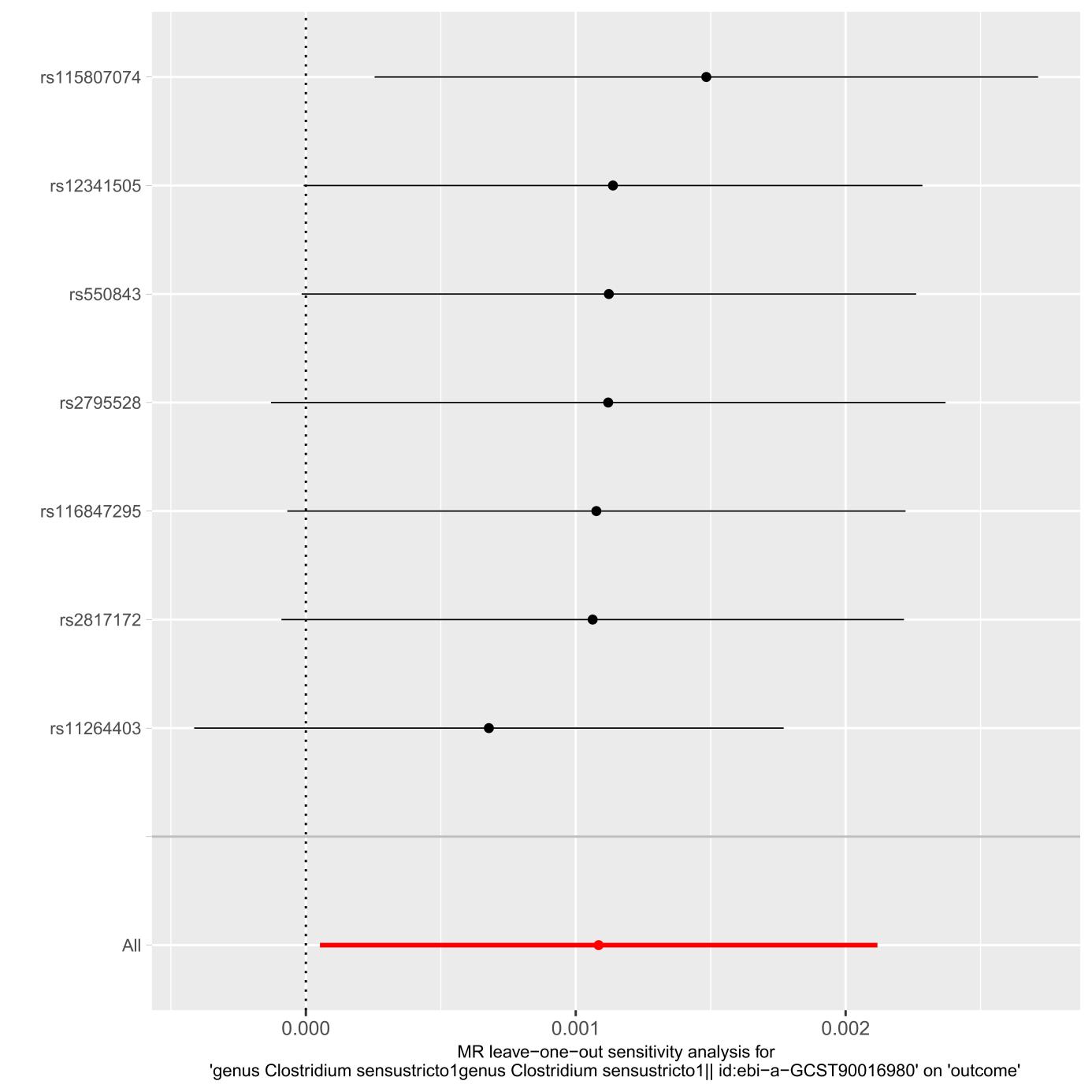

Supplement: Supplementary file 5 [file Image_3.jpg]

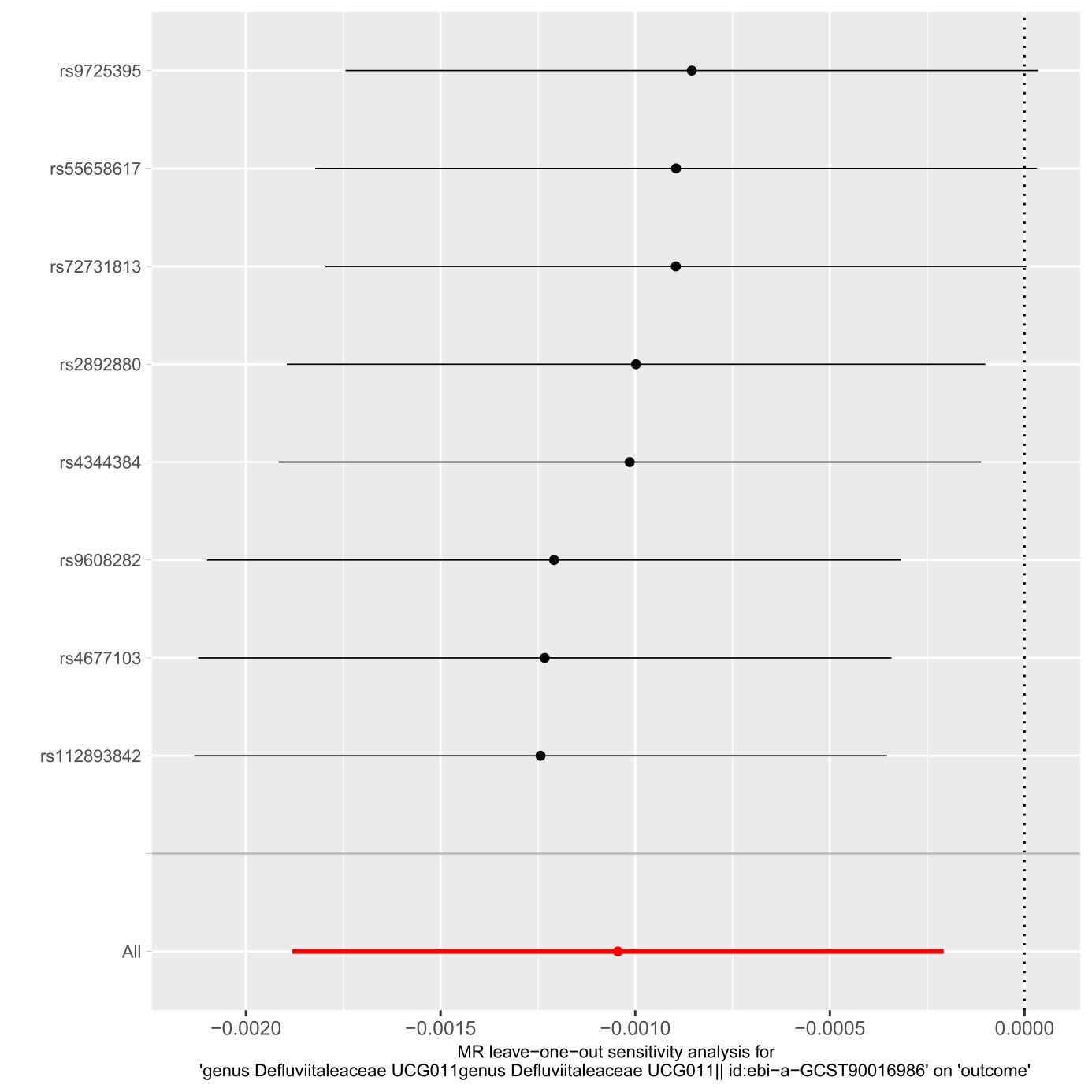

Supplement: Supplementary file 6 [file Image_4.jpg]

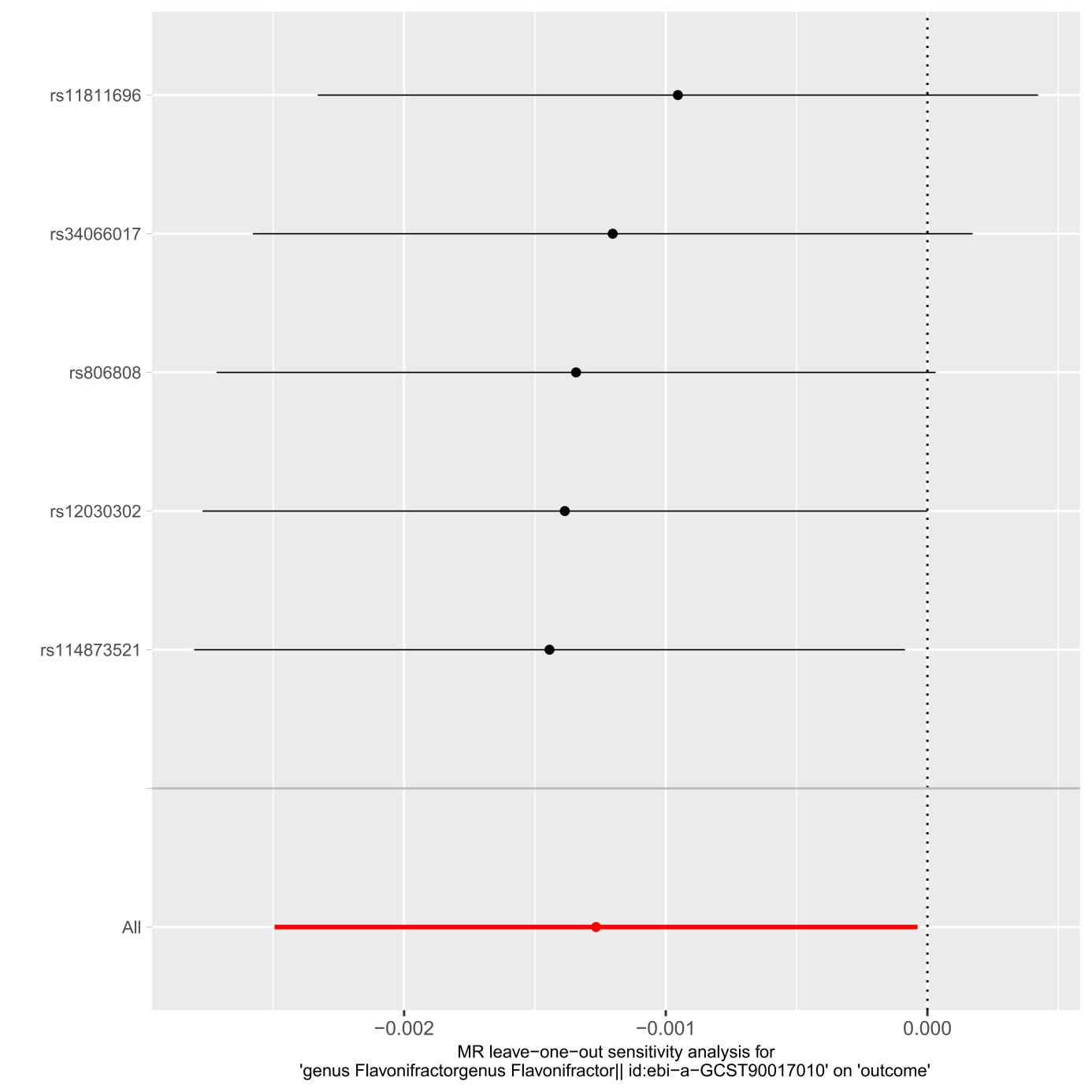

Supplement: Supplementary file 7 [file Image_5.jpg]

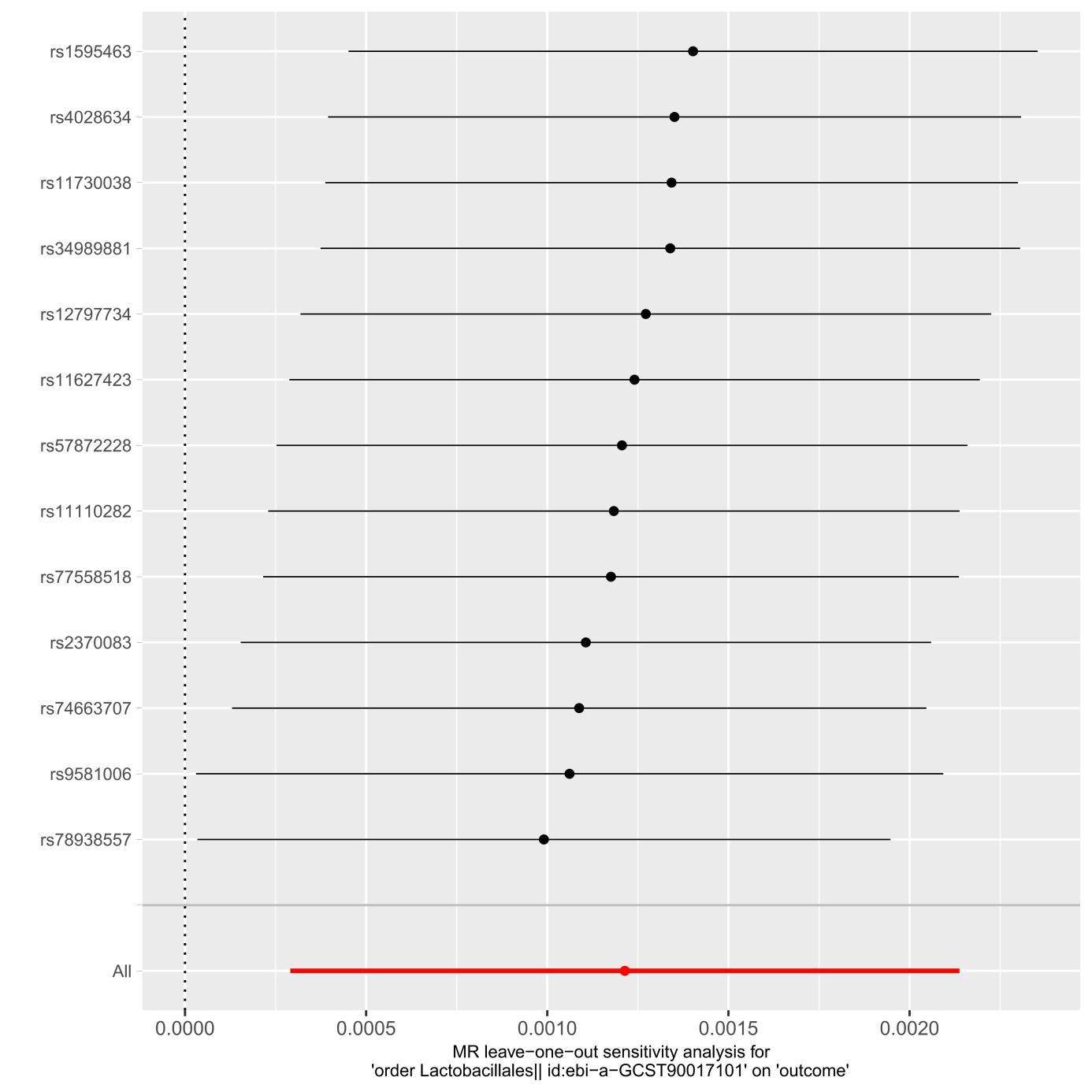

Supplement: Supplementary file 8 [file Image_6.jpg]
